# Supplementary material for: Being a military child in Denmark: Young people’s experiences of living with a parent with PTSD
Source: PLOS Ment Health. 2025 Jan 8;2(1):e0000144. doi: 10.1371/journal.pmen.0000144 (PMC12798285; doi:10.1371/journal.pmen.0000144)
Supplement: Attachment — Submitted filename: SSOP_Email - Paul Watson - Outlook.pdf [file pmen.0000144.s001.pdf]

Re: Confirmation You will receive Revise notification for ethics appllication this simply to upload SSOP email!

Paul Watson <paul5.watson@northumbria.ac.uk>

Thu 01/08/2019 07:37

To:Catherine Bailey <catherine.bailey@northumbria.ac.uk>

Good morning Cathy

I have put the following documents together in one document

Risk assessments 1854 & 7365
Travel Risk assessment PW & AO

I have also attached:

SSOP declaration
Participants information

The PDF file remains the same, with the remaining documents as reviewed previously.

Thank you again so much for taking the time to see this through on your days off. I am so grateful.

BW

From: Paul Watson
Sent: 31 July 2019 20:39:28
To: Catherine Bailey <catherine.bailey@northumbria.ac.uk>
Subject: Re: Confirmation You will receive Revise notification for ethics appllication this simply to upload SSOP email!

Brilliant.

I have uploaded the documentation now.

BW

Paul Watson BSc (MH), PgDip (SCPHN)

Lecturer, Department of Nursing,Midwifery & Health
PhD Candidate - Military Young Carers
Ambassador to The Baton

T: 0191 2156129
E: Paul5.Watson@northumria.ac.uk
Room H216, Coach Lane Campus (East), Northumbria University, Newcastle upon Tyne, NE7 7XA, United Kingdom

---

From: Catherine Bailey <catherine.bailey@northumbria.ac.uk>
Sent: Wednesday, July 31, 2019 8:26:55 PM
To: Paul Watson <paul5.watson@northumbria.ac.uk>
Subject: Re: Confirmation You will receive Revise notification for ethics appllication this simply to upload SSOP email!

Sorry Paul no need to copy Nick in now!

Cheers
Cathy

From: Catherine Bailey <catherine.bailey@northumbria.ac.uk>
Sent: 31 July 2019 20:23:39
To: Paul Watson <paul5.watson@northumbria.ac.uk>
Cc: Nick Neave <nick.neave@northumbria.ac.uk>
Subject: Re: Confirmation You will receive Revise notification for ethics appllication this simply to upload SSOP email!

Hopefully once you attach and send back to me I can approve!

Thanks

Cathy

From: Paul Watson <paul5.watson@northumbria.ac.uk>
Sent: 31 July 2019 20:03:25
To: Catherine Bailey <catherine.bailey@northumbria.ac.uk>
Cc: Nick Neave <nick.neave@northumbria.ac.uk>
Subject: Re: Confirmation You will receive Revise notification for ethics appllication this simply to upload SSOP email!

Hi Cathy

Thank you so much.

I have completed this now so there is space to upload.

BW

From: Catherine Bailey <catherine.bailey@northumbria.ac.uk>
Sent: 31 July 2019 19:47:17
To: Paul Watson <paul5.watson@northumbria.ac.uk>
Cc: Nick Neave <nick.neave@northumbria.ac.uk>
Subject: Confirmation You will receive Revise notification for ethics appllication this simply to upload SSOP email!

Dear Paul

You will receive a 'revise' notification for your ethics application - please see my comment under Title and Objectives section - this is simply so that you can upload the SSOP email as below - in response to our request for written confirmation from this organisation re they having processes and docuimentation in place for consent and assent procedures and as discussed.

I was unable to upload this for you as you already have the maximum number of documents (5) uploaded. Please can you collapse a couple of these into one and then you should be able to attach the email (copied into workd document is fine)

Please submit asap and then I will approve!!

Very best wishes

Cathy

From: Catherine Bailey
Sent: 31 July 2019 18:09
To: Paul Watson <paul5.watson@northumbria.ac.uk>
Cc: Nick Neave <nick.neave@northumbria.ac.uk>
Subject: Re: Confirmation

Dear Paul

Thank you for taking my call earlier. As explained I sought the advice of our Faculty Ethics Director, Prof Nick Neave cc'd here and we both agreed that we needed written confirmation from the Danish organisation that you will be working with, that they have in place, relevant consent, assent documentation including participant information.

Many thanks for acting on this request so promptly Paul re the email from SSOP below.

As ethics coordinator I will record this request and response within your ethics-online application so that we can issue an approval outcome.

Best wishes

Cathy

From: Paul Watson <paul5.watson@northumbria.ac.uk>
Sent: 31 July 2019 12:48
To: Catherine Bailey <catherine.bailey@northumbria.ac.uk>
Subject: Fw: Confirmation

Good afternoon Cathy

As discussed please find attached the email from Bente confirming that SSOP have taken responsibility for consent and assent.

Thank you again for your support it is very much appreciated.

BW

From: paul.watson <p.watson@northumbria.ac.uk>
Sent: 31 July 2019 12:44
To: Paul Watson <paul5.watson@northumbria.ac.uk>
Subject: Fwd: Confirmation

Paul Watson BSc (MH), PgDip (SCPHN)

Lecturer, Department of Nursing,Midwifery & Health
PhD Candidate - Military Young Carers
Ambassador to The Baton

T: 0191 2156129
E: Paul5.Watson@northumria.ac.uk
Room H216, Coach Lane Campus (East), Northumbria University, Newcastle upon Tyne, NE7 7XA, United Kingdom

---

From: Bente Holm Skov <Bente@skovconsult.dk>
Sent: Wednesday, July 31, 2019 12:23:14 PM
To: paul.watson <p.watson@northumbria.ac.uk>
Cc: Lotte.Kolind@mail.dk <Lotte.Kolind@mail.dk>
Subject: Confirmation

Paul,

As agreed I can confirm, that

- SSOP have sent and will receive the consent documentation from the parents of the young people wanting to take part in the research evaluation.

- SSOP have sent and will receive the assent documentation from the young people 16 years plus, wanting to take part in the research evaluation.

- SSOP is a well-established charity, with extensive experience in supporting this population of young people's emotional health and well-being.

On behalf of SSOP

Bente Holm Skov
Chairman for the Charity Commitee and Project Manager for The Invisible Children Project

Email: bente@skovconsult.dk
Tlf.: +455364 4353
www.ssop.dk

http://www.ssop.dk/images/uploads/layout/ssop_logo.gif
