# Supplementary material for: Being a military child in Denmark: Young people’s experiences of living with a parent with PTSD
Source: PLOS Ment Health. 2025 Jan 8;2(1):e0000144. doi: 10.1371/journal.pmen.0000144 (PMC12798285; doi:10.1371/journal.pmen.0000144)
Supplement: Attachment — Submitted filename: Response_to_Reviewers_24.09.03 .docx [file pmen.0000144.s002.docx]

| Section | Review 1 Comment | Author Comment | Line(s) |
| --- | --- | --- | --- |
| Abstract | check - critically evaluate the impact ...during a five-day | Thank you for your comment this has been addressed. | 13-14 |
| Introduction | paragraph (para) line 2 - - stereotyped group - consider (e.g. rebellion) | Thank you for your comment. I have left this as stereotype as this is what is recorded in the wider literature. |  |
|  | This includes surviving serious injuries due to improved weaponry....alongside enhanced rehabilitation. | Thank you for your comment this has been addressed. | 36 |
| Method | What is SSOP - a local charity? | Thank you for your comment this has been addressed. | 93 |
|  | The impact of worrying ... forward forecasting - forecasting perhaps  and always being .... the 2nd part of the sentence can be reworded to flow better. | Thank you for your comment this has been addressed. | 228-229 |
| Results | 4th quote - repetition from page 5 (bottom page) | Thank you for your comment this has been removed. | 233-234 |
| Discussion | sense of cohesion ...could add (and separation?) | Thank you for your comment this has been addressed. | 323 |
|  | Research - r | Thank you for your comment this has been addressed. | 326 |
|  | missing comma after study | Thank you for your comment this has been addressed. | 331 |
|  | parent's ability | Thank you for your comment this has been addressed. | 336 |
|  | Never Dad and is something | Thank you for your comment this has been addressed. | 350-352 |

| Section | Review 2 Comment | Author Comment | Line(s) |
| --- | --- | --- | --- |
| Abstract | Moreover, this article, aims to understand the lived experiences of children and young people from military families and to | Thank you for your comment ‘connected’ has been removed. | 10 |
|  | Also please modify the abstract to clarify that the present paper did not evaluate the impact of the residential camp on the interview subjects. | Thank you for your comment this has been addressed. | 12-15 |
| Methodology | Ethics | Thank you for your comment this has been addressed. | 88-90 |
| Results | He always smiled, but now he is like, he still comes into us, but that wink is not there. | Thank you for your comment this has been addressed | 140 |
| General | The paper could benefit from a clearly marked limitations section. | Thank you for your comment this has been addressed. | 385 |
|  | The paper could greatly benefit from an elaboration on the types and frequency of self-harm these youth have engaged in. | Thank you for your comment this has been addressed. The frequency is mentioned on line 253 but is a general comment as we did not measure overall frequency, so I am unable to comment on this further. | 212 |
|  | The authors mention “PTSD has been described as a debilitating disorder which affects the person with PTSD and those who are close to them.” The paper could benefit from providing examples to the debilitating effects of PTSD. E.g. what are the reasons the interviewees are in the center, or what are some of their experiences that could be described as debilitating, rather than to referring to literature. Also, some of these examples could be given in case of family members of the children. | Thank you for your comment this has been addressed. | 396-405 |
