# Supplementary material for: Being a military child in Denmark: Young people’s experiences of living with a parent with PTSD
Source: PLOS Ment Health. 2025 Jan 8;2(1):e0000144. doi: 10.1371/journal.pmen.0000144 (PMC12798285; doi:10.1371/journal.pmen.0000144)
Supplement: Attachment — Submitted filename: Response_to_Reviewers_24.11.14.docx [file pmen.0000144.s004.docx]

|  | Review 3 Comment | Author Comment | Line(s) |
| --- | --- | --- | --- |
| Abstract | Remove "of" to read "the impact during a five-day residential camp." | Thank you for your comment this has been addressed. | 13 |
| Introduction | Please reword the following sentence for clarity and precision: "Due to recent conflicts military personnel, their families and veterans face different physical and psychological issues." Suggestion: "Recent conflicts have led military personnel, their families, and veterans to face a variety of physical and psychological challenges." | Thank you for your comment this has been addressed. | 44 |
|  | Since the study focuses on Danish military families, could you provide more specific information about Denmark’s military engagements in terms of the unique aspects of Danish military culture, and any support systems in place for military families. | Thank you for your comment this has been addressed. | 28-38 |
| Method | please could you include a statement of reflexivity, bias and your philosophical underpinning. | Thank you for your comment this has been addressed. | 98-104 |
| Results | The final sentence in the first paragraph on page six, “Importantly, a number of the participants’ parents had left the military before they were born, demonstrating the potential long-term effects of military deployment to a combat zone,” seems redundant amongst the quote that follows. However, the first sentence in the paragraph that follows “…many participants could see a change in their parent due to the symptomology of PTSD or other adjustment issues, with many participants remembering how their parent was prior to deployment” seems better placed in this section instead. | Thank you for your comment this has been addressed. | 152-154 |
|  | Could the following sentence on the bottom of p7: “Within the study, participants focussed less on the wider impact their mother’s poor mental health had on themselves, or the rest of their family, providing a more descriptive reality to their home environment” be re-worded to: "In the study, participants focused less on how their mother's poor mental health impacted themselves or the rest of their family, and instead provided more descriptive accounts of their home environment” for better clarity. | Thank you for your comment this has been addressed. | 201-202 |
|  | It appears that some themes have subthemes while others do not. To maintain consistency, it may be clearer to remove the subthemes and retain only the overarching themes. Additionally, the term 'conflict' is used both to refer to a specific foreign conflict (in brackets) and as a subtheme to describe 'conflict' within families. Based on the quotes, this seems to refer more to emotional conflict within the family, so clarifying this distinction would be helpful. However, as mentioned in my earlier comment, removing subthemes altogether may provide a more streamlined structure and avoid any inconsistencies. | Thank you for your comment this has been addressed and I have removed sub themes. |  |
|  | Please ensure that there is no more than two quotes between each descriptive section. | Thank you for your comment this has been addressed. |  |
|  | The end of the results section would benefit from a central organising concept that unifies the key themes – military life and deployment, parental mental health, young people’s emotional health and wellbeing and connectedness – into an overarching narrative. It would help to illustrate the interconnected challenges faced by children from military families and emphasise the broader implications for support and intervention. | Thank you for your comment this has been addressed. | 335-345 |
|  | Please incorporate more quotes related to the theme of self-harm. | Thank you for your comment this has been addressed and I have extended the quote. | 270-271 |
|  | Remove any duplicate quotes and replace them, as the same quote should not be used across different themes. For example, under "Military Life and Deployment," the quote reads: “I miss him a lot. Something is missing at home, we have a big house, and when someone is missing it’s like empty.” However, the same quote is partially repeated, with additional context, under "Young People’s Emotional Health and Well-Being": “I miss him a lot. Something is missing at home, we have a big house, and when someone is missing it’s like empty. Kind of sad, something that is not there, which is normally there. It's tiresome, no matter how sad I am I know he won’t be home, so I try to not think about it, so I am not sad.” | Thank you for your comment this has been addressed and duplicated quotes have been removed. |  |
|  | This section could benefit from a quote to clarify its message: These presentations of feelings of sadness had a wider impact. Specifically, it created emotional conflict for the participants due to a perceived façade they upheld. This façade allowed them to cope with the complexities of their day-to-day lives and interactions with others, to avoid creating additional concerns for their family and their wider network but created a disconnect. | Thank you for your comment this has been addressed. | 259-260 |
| Discussion | Your discussion is really comprehensive and engaging. The existing literature is both relevant and effectively highlights the importance of the study. I found the section on the impact of deployment on family cohesion and the challenges of re-integration particularly insightful and highly relevant to your paper. To strengthen this section further, consider adding a paragraph on the practical and policy implications of your findings, offering specific recommendations for interventions, support systems, or policy changes. For instance, you might suggest how schools or military support services could address issues such as loneliness and mental health among military-connected children. | Thank you for your comment this has been addressed. | 414-426 |
|  | Whilst limitations are acknowledged, it would be beneficial to include more detail on the study's strengths and future directions, particularly with respect to areas for further research (e.g., longitudinal studies, comparisons across military populations in different countries, etc.). | Thank you for your comment this has been addressed. | 435-444 |
| Conclusion | The conclusion effectively summarises the key points, though it could be made more impactful by reiterating the significance of the study’s contributions. Emphasising the need for prevention, early intervention, and continued support for military families affected by PTSD would reinforce the importance of the findings. | Thank you for your comment this has been addressed. | 464-476 |

May I take this opportunity to thank you for your kind words and guidance in reviewing this paper, your mentorship is very much appreciated.
